# Intermittent Preventive Treatment of Malaria Provides Substantial Protection against Malaria in Children Already Protected by an Insecticide-Treated Bednet in Burkina Faso: A Randomised, Double-Blind, Placebo-Controlled Trial

**DOI:** 10.1371/journal.pmed.1000408

**Published:** 2011-02-01

**Authors:** Amadou T. Konaté, Jean Baptiste Yaro, Amidou Z. Ouédraogo, Amidou Diarra, Adama Gansané, Issiaka Soulama, David T. Kangoyé, Youssouf Kaboré, Espérance Ouédraogo, Alphonse Ouédraogo, Alfred B. Tiono, Issa N. Ouédraogo, Daniel Chandramohan, Simon Cousens, Paul J. Milligan, Sodiomon B. Sirima, Brian Greenwood, Diadier A. Diallo

**Affiliations:** 1 Centre National de Recherche et de Formation sur le Paludisme, Ouagadougou, Burkina Faso; 2 London School of Hygiene & Tropical Medicine, London, United Kingdom; University of Melbourne, Australia

## Abstract

**Background:**

Intermittent preventive treatment of malaria in children (IPTc) is a promising new approach to the control of malaria in areas of seasonal malaria transmission but it is not known if IPTc adds to the protection provided by an insecticide-treated net (ITN).

**Methods and Findings:**

An individually randomised, double-blind, placebo-controlled trial of seasonal IPTc was conducted in Burkina Faso in children aged 3 to 59 months who were provided with a long-lasting insecticide-treated bednet (LLIN). Three rounds of treatment with sulphadoxine pyrimethamine plus amodiaquine or placebos were given at monthly intervals during the malaria transmission season. Passive surveillance for malaria episodes was established, a cross-sectional survey was conducted at the end of the malaria transmission season, and use of ITNs was monitored during the intervention period. Incidence rates of malaria were compared using a Cox regression model and generalized linear models were fitted to examine the effect of IPTc on the prevalence of malaria infection, anaemia, and on anthropometric indicators. 3,052 children were screened and 3,014 were enrolled in the trial; 1,505 in the control arm and 1,509 in the intervention arm. Similar proportions of children in the two treatment arms were reported to sleep under an LLIN during the intervention period (93%). The incidence of malaria, defined as fever or history of fever with parasitaemia ≥5,000/µl, was 2.88 (95% confidence interval [CI] 2.70–3.06) per child during the intervention period in the control arm versus 0.87 (95% CI 0.78–0.97) in the intervention arm, a protective efficacy (PE) of 70% (95% CI 66%–74%) (*p<*0.001). There was a 69% (95% CI 6%–90%) reduction in incidence of severe malaria (*p = *0.04) and a 46% (95% CI 7%–69%) (*p = *0.03) reduction in the incidence of all-cause hospital admissions. IPTc reduced the prevalence of malaria infection at the end of the malaria transmission season by 73% (95% CI 68%–77%) (*p<*0.001) and that of moderately severe anaemia by 56% (95% CI 36%–70%) (*p<*0.001). IPTc reduced the risks of wasting (risk ratio [RR]* = *0.79; 95% CI 0.65–1.00) (*p = *0.05) and of being underweight (RR* = *0.84; 95% CI 0.72–0.99) (*p = *0.03). Children who received IPTc were 2.8 (95% CI 2.3–3.5) (*p<*0.001) times more likely to vomit than children who received placebo but no drug-related serious adverse event was recorded.

**Conclusions:**

IPT of malaria provides substantial protection against malaria in children who sleep under an ITN. There is now strong evidence to support the integration of IPTc into malaria control strategies in areas of seasonal malaria transmission.

**Trial Registration:**

ClinicalTrials.gov NCT00738946

*Please see later in the article for the Editors' Summary*

## Abstract 2

A randomized trial reported by Diadier Diallo and colleagues shows that intermittent preventive treatment for malaria in children who are protected from mosquitoes using insecticide-treated bednets provides substantial protection from malaria.

## Editors' Summary

**Background:**

Malaria accounts for one in five of all childhood deaths in Africa and of the one million annual malarial deaths world-wide, over 75% occur in African children under 5 years old. Malaria also causes severe morbidity in children, such as anemia, low birth weight, and neurological problems, which compromise the health and development of millions of children living in malaria endemic areas. As much of the impact of malaria on African children can be effectively prevented, significant efforts have been made in recent years to improve malaria control, such as the implementation of intermittent preventive treatment of malaria.

Intermittent preventive treatment (IPT) involves administration of antimalarial drugs at defined time intervals to individuals, regardless of whether they are known to be infected with malaria, to prevent morbidity and mortality. IPT was initially recommended for pregnant women and recently this strategy was extended to include infants (IPTi). Now, there is also IPT of malaria in children (IPTc), which is designed to protect against malaria during the high malaria transmission season.

**Why Was This Study Done?:**

Large clinical trials have shown that IPTc involving the administration of two to three doses of an antimalarial drug (sulphadoxine pyrimethamine [SP] and artesunate [AS] or amodiaquine [AQ]) during the high malaria transmission season effectively reduces the incidence of malaria. However, these studies were conducted in countries where the use of insecticide-treated bednets—an intervention that provides at least 50% protection against morbidity from malaria and is the main tool used for malaria control in most of sub-Saharan Africa—was relatively low. Therefore, it is unclear whether IPTc will be as effective in children who sleep under insecticide-treated bednets as has been previously shown in communities where insecticide-treated bednet usage is low. So to determine the answer to this important question, the researchers conducted a randomized, placebo-controlled trial of IPTc with SP + AQ (chosen because of the effectiveness of this combination in a pilot study) in children who slept under an insecticide-treated bednet in an area of seasonal malaria transmission in Burkina Faso.

**What Did the Researchers Do and Find?:**

The researchers enrolled 3,014 eligible children aged 3–59 months into a randomized double-blind, placebo-controlled trial during the 2008 malaria transmission season in Burkina Faso. All children were given a long-lasting insecticide-treated bednet at the start of the study with instructions to their family on the correct use of the net. Children were then randomized into two arms—1,509 were allocated to the intervention group and 1,505 to the control group—to receive three courses of IPTc with SP plus AQ or placebos given at monthly intervals during the peak malaria transmission season. The researchers monitored the incidence of malaria throughout the malaria season and also monitored the use of long-lasting insecticide-treated bednets throughout the study period. In addition, researchers conducted a cross-sectional survey in 150 randomly selected children every week and in every child enrolled in the trial 6 weeks after the last course of IPTc, to measure their temperature, height and weight, and blood hemoglobin and parasite count levels.

The number of children who slept under their long-lasting insecticide-treated bednet was similar in both arms. During the intervention period, the researchers found that the incidence of clinical malaria (defined as fever or a history of fever and the presence of at least 5,000 asexual forms of *P. falciparum* per microliter) was 2.88 in the control arm versus 0.87 in the intervention arm—giving a protective efficacy of 70%. There were 13 cases of severe malaria in the control arm and four in the IPTc arm—a 69% reduction in incidence. Additionally, all-cause hospital admission rate was reduced by 46%. At the end of the malaria transmission period, IPTc reduced the proportion of children infected with malaria parasites by 73% and reduced anemia by 33%. In addition, IPTc appeared to reduce the risk of wasting (risk ratio  = 0.79) and of being underweight (risk ratio  = 0.84). However, children who received IPTc were almost three times more likely to vomit than children who received placebo but there were no drug-related serious adverse events.

**What Do These Findings Mean?:**

The results of this study show that in peak malarial transmission season in Burkina Faso, IPTc provides substantial additional protection against episodes of clinical malaria, severe malaria, and all-cause hospital admissions in children sleeping under long-lasting insecticide-treated bednets. In addition, intermittent preventive treatment of malaria with SP plus AQ appears to be safe for use in children.

**Additional Information:**

Please access these websites via the online version of this summary at http://dx.doi.org/10.1371/journal.pmed.1000408.

- This topic is further discussed in two *PLoS Medicine* research articles: Dicko et al. and Bojang et al., and in a *PLoS Medicine* Perspective by Beeson
- Roll Back Malaria has information about malaria in children, including intervention strategies
- UNICEF also provides comprehensive information about malaria in children
- The Intermittent Preventive Treatment in Infants Consortium (ipti) provides information on intermittent preventive treatment in infants
- Roll Back Malaria has an information sheet on insecticide-treated bednets

## Introduction

Significant efforts have been made in recent years to improve malaria control. However, malaria still remains a major public health problem in sub-Saharan Africa, responsible for about 800,000 deaths annually [1], and existing malaria control strategies provide only partial protection. The pressing need for new malaria control tools has led to evaluation of the strategy of intermittent preventive treatment (IPT) of malaria. IPT involves administration of antimalarial drugs at defined time intervals to individuals regardless of whether they are known to be infected with malaria to prevent morbidity and mortality from the infection [2]. IPT was initially recommended for pregnant women involving the administration of at least two doses of sulphadoxine pyrimethamine (SP) during antenatal visits after the first trimester of pregnancy. Recently the strategy was extended to infants (IPTi) with the administration of three doses of an antimalarial drug during the expanded programme of immunization (EPI) visits [3]. An Institute of Medicine report [4] indicated that IPTi is associated with a 30% (95% confidence interval [CI] 20%–39%) reduction in the incidence of clinical malaria. However in areas of seasonal malaria transmission, such as the Sahel and the sub-Sahelian regions of Africa, the main burden of malaria is in children under 5 y of age [5]. The strategy of IPT of malaria in children (IPTc) was designed for regions where malaria transmission is seasonal [6].

IPTc involves the administration of two to three doses of antimalarial drug during the high malaria transmission season. IPTc with artesunate (AS) plus amodiaquine (AQ) given to children aged 3–59 mo old on three occasions at monthly intervals during the malaria transmission season led to an 86% reduction in the incidence of clinical episodes of malaria in Senegal [6]. In an earlier study conducted in Mali, IPTc with sulphadoxine pyrimethamine (SP), administered on two occasions during the malaria transmission season with a 2-mo interval between treatments, reduced the incidence of malaria in children aged 6 mo to 10 y by 65% [7]. Similar findings were reported in Ghana in an area with perennial malaria transmission but a major seasonal peak, where six monthly rounds of administration of AS+AQ led to a 69% decrease in the incidence of malaria in children [8]. IPTc was safe and well tolerated in each of these studies [Bibr pmed.1000408-Ciss1] [Bibr pmed.1000408-Dicko1] [6–8], and no evidence of a rebound in the incidence of malaria was observed in the year after the intervention was stopped [6].

Insecticide-treated bednets (ITNs) provide at least 50% protection against morbidity from malaria [9] and are currently the cornerstone of malaria control in many countries in sub-Saharan Africa, although coverage with ITNs is still low in some countries. For example, in Burkina Faso it was estimated that between 2003 and 2006, fewer than 20% of households owned an ITN and less than 10% of children aged below 5 y of age slept under an ITN [1]. However, strenuous efforts are being made to increase coverage in endemic areas and in Burkina Faso, the National Malaria Control Programme (NMCP) has started procedures to purchase about 6 million long-lasting insecticide-treated bednets (LLINs).

The successful trials of IPTc described above were conducted in areas with relatively low coverage of ITNs. Thus, it is not known whether IPTc will be as effective in children who sleep under an ITN as has been found in communities where ITN usage is low. To determine this difference, we have conducted a randomised, placebo-controlled trial of IPTc with SP + AQ in children who slept under an ITN in an area of seasonal malaria transmission in Burkina Faso. A parallel study has been conducted in Mali employing a very similar protocol [10], and it was planned to involve a third site from Ghana. However, this site could not participate because of delays in getting regulatory approval for the use of SP + AQ for IPTc from the Ghana Food and Drug Board.

SP + AQ combination was chosen because this drug combination is cheap and remains highly efficacious for malaria treatment in Burkina Faso [Bibr pmed.1000408-Zongo1] [11,12]. SP was considered as a second-line drug for the treatment of malaria before the introduction of artermisinin-based combination therapy, but the drug was seldom used, as was AQ. A previous study in Senegal showed that the SP + AQ combination was very effective for reducing the incidence of clinical malaria when used for IPT in children [13]. However, the main concern with this drug combination is the increased risk of vomiting associated with AQ.

## Materials and Methods

The protocol of the trial (Text S1), protocol amendment (Text S2), and CONSORT checklist (Text S3) are available as supporting information.

### Study Design

An individually randomised, double-blind, placebo-controlled trial was carried out during the 2008 malaria transmission season to evaluate the efficacy of IPTc in children who slept under an LLIN. All children enrolled in the trial (aged 3–59 mo) were given an LLIN at the start of the study and their family instructed in the use of the net. Children were then randomised to receive three courses of IPTc with SP plus AQ or placebos given at monthly intervals during the peak malaria transmission season. The combination of SP plus AQ was chosen for the trial following a pilot study conducted in the study villages in 2006, which showed that this combination was effective at treating uncomplicated falciparum malaria (Text S4). The incidence of malaria was monitored throughout the malaria season and a cross-sectional survey was performed at its end.

### Ethical Approval

Ethical clearance for the trial was obtained from the Health Ethics Committee of Burkina Faso and from the London School of Hygiene and Tropical Medicine ethics committee. The study's objectives and methods were explained to each study community prior to the start of the trial and written informed consent was obtained from care givers of children before enrolment in the study. The trial was monitored by an independent Data Safety and Monitoring Board (DSMB).

### Study Area

The study was carried out in four villages (Laye, Niou, Sao, and Toeghin) in Boussé health district, Kourweogo province, approximately 40 km northwest of Ouagadougou. The climate in the study area is characteristic of the Sudanese savannah with a dry season from November to June and a rainy season from July to October. The main malaria vectors are *Anopheles gambiae* s.s., *An. Arabiensis*, and *An. funestus* [14]. Malaria transmission is high but seasonal. In 2002, the entomological inoculation rate (EIR) was estimated to be 173 infective bites per person per year [15] with a peak in September. *Plasmodium falciparum* is responsible for more than 95% of malaria infections in the study area.

### Study Endpoints

The primary endpoint of the study was the incidence of clinical malaria defined as the presence of fever (axillary temperature ≥37.5°C) or a history of fever in the past 24 h, the absence of any other obvious cause of fever, and the presence of at least 5,000 asexual parasites of *P. falciparum* per microlitre. This threshold has previously been shown to be of value in differentiating symptomatic malaria from other causes of fever with coincidental parasitaemia [16]. The secondary endpoints were: (1) the incidence of clinical malaria defined as the presence of fever or history of fever, the absence of any other obvious cause of fever, and the presence of *P. falciparum* asexual parasites at any density; (2) the incidence of severe malaria defined according to WHO criteria [17]; (3) the prevalence of anaemia at the end of malaria transmission season; (4) the prevalence of parasitaemia at the end of the malaria transmission season; (5) the prevalence of wasting, stunting, and being underweight at the end of malaria transmission season; (6) the incidence of all-cause hospital admissions during the surveillance period.

Hospital admission was defined as any event that involved a child staying at a hospital or health centre for at least 24 h for medical care. Anaemia was defined as a haemoglobin (Hb) concentration <11 g/dl, moderately severe anaemia as an Hb <8 g/dl, and severe anaemia as an Hb <5 g/dl. Wasting (acute malnutrition), stunting (chronic malnutrition), and underweight (chronic and/or acute malnutrition) were assessed using the WHO child growth standards [18]. Children with weight-for-age *z*-scores (WAZ) <−2 were classified as underweight and children with height-for-age *z*-scores (HAZ) <−2 were classified as stunted. Children with weight-for-height *z*-scores (WHZ) <−2 were classified as wasted.

### Sample Size

We first calculated the sample size required to provide the trial with 90% power to detect a 20% reduction in the incidence of clinical malaria in the IPTc arm of the study at the 5% level of significance. We assumed that the incidence of clinical malaria in children under 5 y of age in the study area, in the absence of any intervention, would be in the range of 1.0–2.0 episodes per year. We further assumed that in children protected by an ITN, the incidence of malaria would be reduced by 50% to 0.5–1.0 episodes per year. To detect a 20% reduction in the incidence of malaria, from 0.5 to 0.4 episode per year, in children who received IPTc, and allowing for a 20% loss to follow-up, we estimated that approximately 1,000 children would be required in each study arm. We further estimated that a study with this sample size would have at least 90% power to detect a 20% reduction in the prevalence of malaria infection (from 50% to 40%) at the end of the malaria transmission season [19].

With a sample size of 1,000 children per arm, the study was not powered to detect an impact on the incidence of severe malaria or hospitalization with malaria. We estimated that the rate of hospital admissions with malaria would be in the range of 20 to 60 per 1,000 children under 5 y of age. Assuming a rate of 40 per 1,000, approximately 3,000 children would be required per treatment arm to detect a 50% reduction in hospital admissions with severe malaria (from 40 to 20 per 1,000), assuming 20% loss to follow-up. A protocol amendment was made to increase the sample size after the site in Ghana was dropped from the trial (Text S2). Therefore, the sample size was increased to 1,500 children per treatment arm in our trial and in a parallel study conducted in Mali [10], with the intention of combining results from the two trials to investigate the impact of IPTc on the severe malaria endpoint.

### Enrolment and Randomisation

Study villages were located in an area in which a demographic surveillance system (DSS) was implemented until 2002. We used the DSS database to identify and select four villages, each with a health centre and with a combined population of 3,600 to 3,800 children aged 3–59 mo. The aim was to sample about 500 children from the DSS database for the baseline prevalence of genetic markers of resistance to SP and AQ and screen the remaining children for enrolment in the trial, allowing for the possibility that a fraction of these children would not meet the inclusion criteria. Therefore, all children aged 3–59 mo in the four selected study villages were enumerated before the start of the intervention and screened for eligibility to participate in the study. The child's weight and height were measured. To be eligible, a child had to fulfil the following inclusion criteria: body weight of at least 5 kg, resident in one of the study villages with no plan to move out of the study area during the intervention period, no signs or symptoms of severe chronic illness, absence of signs of severe malnutrition, and signed informed consent obtained from the care giver. Children with a history of sensitivity to any antimalarial drug were excluded. Children diagnosed with clinical malaria during screening were randomised and then treated according to local guidelines.

Eligible children were randomised to the intervention or control group using sealed envelopes. The randomisation list was prepared in three strata for children weighing 5–9 kg, 10–18 kg, and ≥19 kg by a statistician, and treatment group was assigned in each stratum in a 1∶1 ratio in permuted blocks of size 10. Blocks were not split across villages. Envelopes in each stratum were assigned in strict numerical order by the research team. When the child was enrolled, the child's name and randomization code were written on the outside of the envelope before the envelope was opened. Each child was given an identification (ID) card to facilitate follow-up.

### LLINs

All screened children were given an LLIN (PermaNet; www.vestergaard-frandsen.com) regardless of their eligibility status. LLINs were marked to differentiate them from already existing nets and care givers were advised on how to use them.

### Drugs and Packaging

A course of IPTc comprised a single treatment with SP and three daily doses of AQ, the first dose of AQ being given with SP. The target dose for SP was 25 mg of sulphadoxine and 1.25 mg pyrimethamine per kg, and that for AQ was 10 mg per kg daily given for 3 d. Active SP and AQ tablets and matching placebos were obtained from KINAPHARMA Limited. To achieve as accurate a dosing schedule as possible, whilst maintaining simplicity in delivery and avoiding the need to break tablets, three sets of SP and AQ tablets were manufactured. SP tablets for children in the 5–9 kg weight group contained 175 mg of sulphadoxine and 8.75 mg of pyrimethamine per tablet, those for children in the 10–18 kg group contained 350 mg of sulphadoxine and 17.5 mg of pyrimethamine, and those for children 19 kg or more contained 550 mg sulphadoxine and 26.25 mg of pyrimethamine. AQ tablets for children in each of the three weight categories contained daily doses of 70, 140, and 220 mg of AQ, respectively. This regimen gave an AQ dose of 7.8–14 mg/kg/d, which is within the recommended therapeutic range of 7–15 mg/kg/d [20].

Drugs for each course of treatment were packaged in small, sealable plastic bags labelled with a child's randomisation code and course number and placed in an envelope labelled with the child's randomisation code and the village number. A fourth plastic bag containing replacement doses for SP and AQ was included in each envelope. Samples of active and placebo tablets from different containers provided by the manufacturer were analysed at the London School of Hygiene and Tropical Medicine to confirm drug quality before the intervention and to check the accuracy of the randomisation and packing procedure. Drug content and solubility met international standards.

### Intervention

In each village, IPTc was administered at a convenient location close to the health centre and supervised by the research team. The research team and the care givers were blinded as to which group of children received active drugs or placebos. Treatment courses were given in August, September, and October during the peak malaria transmission season, with 1 mo intervals between treatments. Active drugs and placebos were crushed and mixed with sugary water to mask the bitterness of AQ and to improve drug intake. Children were observed for 30 min after treatment. If a child vomited or regurgitated the drug within this period, a second treatment was given. If the child vomited the second treatment no further replacement dose was given for that treatment course but the child was asked to attend the next day or the next round of treatment.

Children were examined before administration of the first dose of each treatment round and a rapid diagnostic test (RDT; OPTIMAL IT; Diamed AG) for malaria was performed for children with fever or a history of fever in the previous 24 h. If the RDT was negative, the child was given IPTc and appointments were made for subsequent IPTc visits before referral to the health centre to check for illnesses other than malaria. If the RDT was positive, a blood film was prepared and the child was treated with Coartem by the research team; children with severe malaria were referred to the health centres for treatment with quinine according to local guidelines. In such situations, IPTc was not given but the care giver was invited to bring the child back for the next round of IPTc.

### Passive Surveillance for Clinical Malaria

Passive surveillance for malaria and other illnesses was established soon after the enrolment of children. Health centres were supplied with lists of children enrolled in the study and with equipment and consumables for the diagnosis of malaria and anaemia. Care givers were asked to bring any child with fever to the health centre together with the child's ID card. The child's identity was verified against the list of enrolled children. Clinical examination was then conducted and signs and symptoms of any illness were recorded. An RDT was performed and if the test was positive, thick and thin blood films were prepared and sent to the CNRFP laboratory for microscopy. Coartem was not available at the health centres, so on the basis of RDT test results, treatment with AS+AQ was given if the child was suspected to have uncomplicated malaria. If a child had signs or symptoms of severe malaria, s/he was treated with quinine at the health centre or referred to the district hospital according to local guidelines. Children whose RDT was negative were examined for other causes of fever and an appropriate treatment was administered if required. A medically qualified member of the research team was based at the district hospital with responsibility for follow-up of study children who were referred to the district hospital. Full treatment costs, and the cost of transportation to the district hospital, were covered by the project.

### Monitoring of Malaria Infection, Anaemia, and Anthropometric Indicators

From mid-September to the end of November 2008, 150 enrolled children were randomly selected and surveyed each week to monitor the prevalence of malaria infection. These children were visited at home by nurses who measured the child's temperature, prepared thick and thin blood films for all children, and completed data collection forms. Care givers were asked if the child had had a fever within the last 24 h and whether the LLIN allocated to them was still in the house. If the LLIN was present, the care giver was asked if the child had slept under the net the previous night. Children with measured or reported fever were referred to the health centre for clinical examination and treatment as described above.

A cross-sectional survey of all children enrolled in the study was carried out 6 wk after the last course of IPTc had been given (November 2008). Fever within the last 24 h was documented, and axillary temperature, weight, and height were measured. Thick and thin blood films and filter paper blood spots were prepared and Hb concentration was measured. An RDT was performed if a child had fever or a history of fever before referral to the health centre for clinical examination and appropriate treatment.

### Monitoring of Adverse Events

Clinical personnel participating in the trial were trained to identify serious adverse events, including Stevens-Johnson syndrome, and to report them immediately to the local principal investigator. Adverse events were monitored on the day of administration of each dose and on the day after the last dose of each treatment course by members of the research team who were not involved in giving treatment. Questions were asked specifically about the occurrence of any of the following adverse events: fever, vomiting, diarrhoea, drowsiness, skin rash, coughing, loss of appetite, and any other events that the child had experienced were recorded. The duration and severity of adverse events was noted. If a serious adverse event was observed or reported, the Data Safety and Monitoring Board (DSMB) was notified of this event within 72 h. Detailed information was collected on a separate case report form and sent to the DSMB within 2 wk.

### Monitoring of Drug Resistance

412 children not included in the main trial were randomly selected from the census list to assess the baseline prevalence of genetic markers of resistance to SP and AQ. Children were clinically examined, and thick and thin blood films and filter paper blood spots were prepared for malaria diagnosis and molecular testing for drug resistance. These children were given an LLIN but were not enrolled in the IPTc trial. Filter paper blood spots were also collected 6 wk after the third course of treatment from children involved in the trial to assess whether treatment with SP + AQ had led to an increase in the prevalence of genetic markers of resistance to these drugs. 312 of the filter papers collected from 746 children with *P. falciparum* parasitaemia during this survey were randomly selected from the intervention and control arms for PCR analysis. This sample size was based on results obtained from the monitoring of resistance markers in an IPT trial in Senegalese children [6]. In addition to monitoring the prevalence of genetic markers of resistance to the study drugs, an in vivo study of the efficacy of SP + AQ combination was undertaken in 252 children with asymptomatic malaria in the year after the intervention (Text S5).

### Laboratory Methods

Thick and thin blood films were air dried and stained with 5% Giemsa. Each slide was read by two laboratory technicians. Asexual and sexual parasites were counted separately and species differentiated. Malaria parasites were counted against 200 white blood cells (WBC). A slide was declared negative only after reading against 2,000 WBC without observation of a malaria parasite. The parasite count was converted to a parasite density per microlitre of blood by multiplying the number of parasites counted by 8,000 and dividing by the number of WBC counted. In the event of a discrepancy between the two readers in terms of the presence or absence of malaria parasites, or if parasite densities differed by more than 30%, the slide was reexamined by a third laboratory technician. The arithmetic mean of the two final readings was used as the final parasite density. If there was no agreement after the third reading, the arithmetic mean of the three parasite densities was used. External quality control of slide reading was performed by the Malaria Diagnosis Centre of Excellence (MDCoE) of the Walter Reed/Kenya Medical Research Institute, in Kisumu (Kenya). The results showed that the quality of slide reading was good with higher than 90% concordance on parasite detection and species identification (Text S6).

Hb concentration was determined using a Hemocue 321 (Hemocue AB). The measurement was repeated for any Hb value outside the manufacturer's range (<5 g/dl and >18 g/dl). The performance of the Hemocue was checked weekly with samples of known Hb concentration.

DNA was extracted from filter papers collected before and after the intervention to look for the presence of genetic markers of resistance to SP and AQ. Nested PCR was performed to detect the presence of mutations at codons 51, 59, and 108 of the *dhfr* gene and at codons 437 and 540 of the *dhps* gene as described previously [21]. Restriction fragment length polymorphism was used to determine the presence or absence of mutations at codon 76 of the *P. falciparum* chloroquine transporter gene (*pfcrt*-76) and at codon 86 of the *P. falciparum* multidrug resistance gene one (*pfmdr1*-86) [22].

### Data Handling and Statistical Analysis

Double data entry by two independent data entry clerks was performed. Data were entered using Microsoft Access. Consistency checks and audit trail programs were developed and the database was cleaned and validated before analysis. The trial analysis plan was approved by the DSMB. Data analysis was performed using STATA version 10 (www.stata.com). Analyses were performed on the basis of intention to treat (ITT).

Episodes of uncomplicated or severe malaria that occurred after the first dose of IPTc and within 42 d of the third course of IPTc administration were included in the analysis. The starting dates of episodes of malaria meeting the primary or secondary definitions above were identified. Child-days at risk were calculated and used as the denominator for the estimation of the incidence of malaria. Any child who experienced an episode of malaria and was treated was not considered at risk for the next 21 d, which were deducted from the child's time at risk. Children who migrated, died, were lost to follow-up, or were withdrawn from the study contributed to the denominator up to the date of the event or up to the date when they were seen for the last time by the research team.

The crude incidence rate ratio (IRR) for the effect of IPTc on the incidence of malaria was estimated using Cox regression models with robust standard errors to account for repeated episodes within the same child. In secondary analyses, age, sex, and village were included in the regression model as covariates to obtain adjusted IRRs. Protective efficacy (PE) was derived from the IRR as follows: PE = (1 − IRR) ×100. Kaplan-Meier survival curves were plotted to compare the times to the first episode of malaria between children who received IPTc and those allocated to the placebo arm.

Incidence rates of all-cause hospital admissions were estimated as the number of all hospital admissions divided by the number of child-days at risk computed as described above.

Malaria parasitaemia was defined as the presence of malaria parasites irrespective of the developmental stage or species. The presence or absence of malaria infection was coded as a binary variable. Proportions of children with malaria parasites during weekly visits and at the cross-sectional survey carried out 6 wk after the last dose of IPTc were estimated for placebo and intervention arms. The presence of anaemia or moderately severe anaemia 6 wk after the last dose of IPTc was also coded as a binary variable. The risk ratios (RRs) for malaria infection, anaemia, and moderately severe anaemia among IPTc children compared with controls were estimated using generalized linear models. Age, sex, and village were included as covariates to obtain adjusted RRs. The PE of IPTc was calculated as follows: PE = (1 − RR) ×100.

Anthropometric data collected 6 wk after the last course of IPTc were transferred from STATA version 10 to WHO's anthropometric software [18] to obtain *z*-scores for weight-for-age (WAZ, underweight), height-for-age (HAZ, stunting), and weight-for-height (WHZ, wasting). Wasting, stunting, and being underweight were defined according to the WHO child growth standards [18] and coded as binary variables based on z-scores <−2. Proportions of wasted, stunted, and underweight children were calculated and compared between intervention and control arms by fitting a generalized linear model to estimate crude and adjusted RRs.

## Results

### Baseline Characteristics

**Figure 1:**
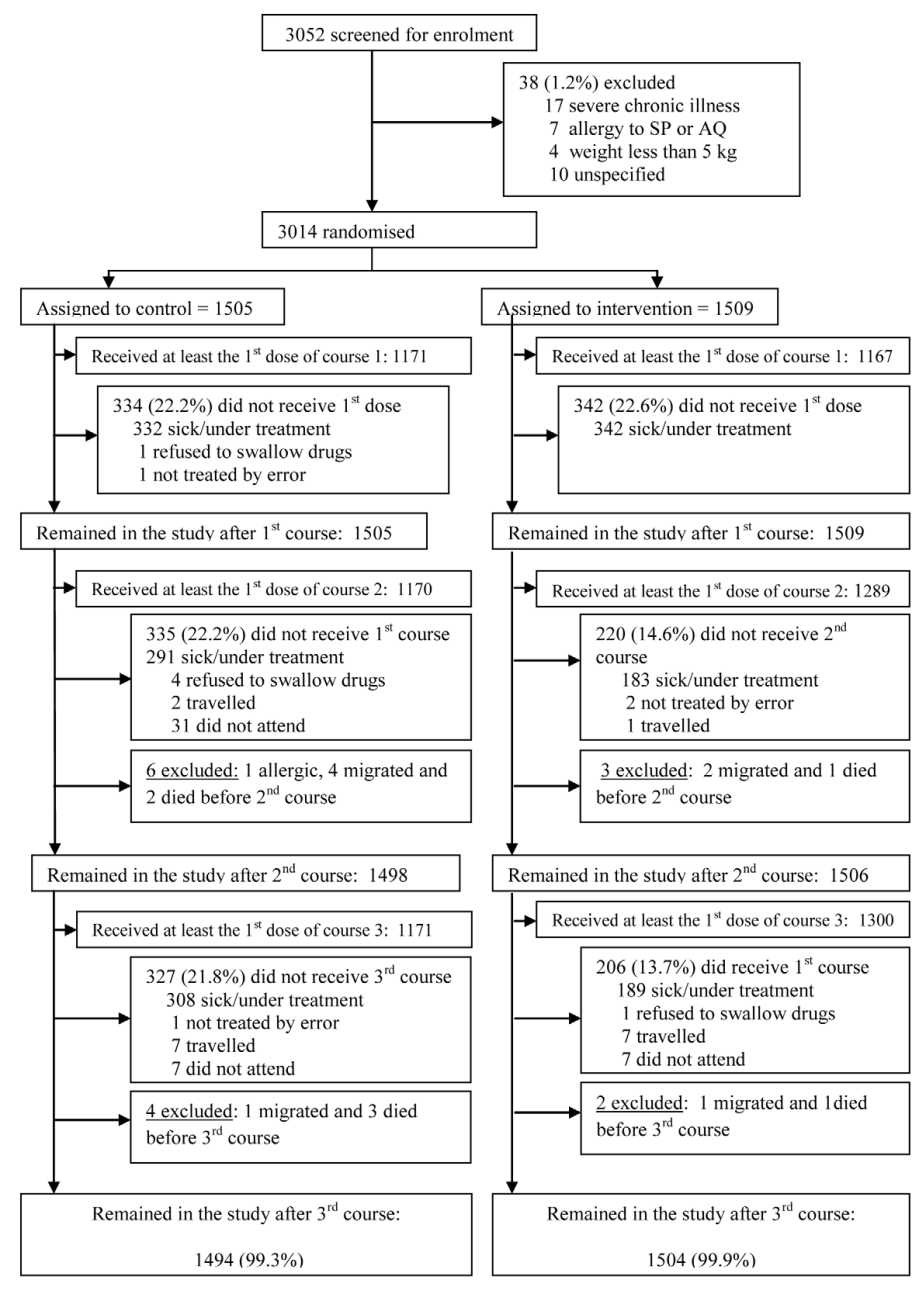
Trial profile.

3,052 children were screened and 3,014 were enrolled in the trial (Figure 1); 1,509 were allocated to the intervention group (SP + AQ) and 1,505 to the control group. The mean age of study children at enrolment was 30.4 mo. The age and sex distributions of children were similar in the intervention and control groups (Table 1), as was their weight distribution. No important differences in the proportions of wasted, stunted, or underweight children at enrolment were observed between the groups. The prevalence of fever at enrolment was also comparable between groups and the proportions of children who used an ITN before the intervention were also similar (less than 0.5%). Similar proportions of children were allocated to the control and intervention groups in each of the four study villages.

**Table 1:** Characteristics of the study children at the time of administration of the first dose of treatment and their use of ITNs before intervention.

Characteristics	Intervention	Control
	Percent (*n*)	Percent (*n*)
	*n = *1,509	*n = *1,505
***Age (mo)***		
3–11	15.3 (231)	16.9 (255)
12–23	23.1 (348)	21.6 (325)
24–35	20.3 (307)	21.0 (316)
36–47	22.6 (341)	20.1 (302)
48–59	18.7 (262)	20.4 (307)
Mean age	30.48 (SD = 15.76)	30.45 (SD = 16.32)
***Sex***		
Male	52.3 (789)	50.5 (760)
Female	47.7 (720)	49.5 (745)
***Weight (kg)***		
5–9	39.3 (593)	39.0 (587)
10–18	60.4 (911)	60.8 (915)
≥19	0.3 (5)	0.2 (3)
Mean weight	11.05 (SD = 3.01)	11.03 (SD = 3.07)
***Nutritional factors***	*n* = 1,496	*n* = 1,488
Wasting	11.9 (178)	11.4 (170)
Stunting	38.9 (582)	38.3 (570)
Underweight	24.6 (368)	25.3 (376)
***Fever***	20.0 (302)	19.9 (299)
***Use of bednets***	*n = *1,428	*n = *1,421
Any net	0.6 (18)	0.8 (11)
Treated net	0.2 (3)	0.5 (7)

SD, standard deviation.

### Treatment and Follow-up

Similar proportions of children in the intervention and control groups received at least the first dose of the first treatment course (77% versus 78%). However, a higher proportion of children in the intervention than in the control group received at least the first dose of the second and the third courses of treatment (85% versus 78% and 86% versus 78%, respectively). The main reason for not receiving treatment was an acute illness. The numbers of children who received a full course of treatment (three doses) at each round were 1,055 (70%), 1,102 (73%), and 1,104 (73%) in the control group and 1,084 (72%), 1,213 (80%), and 1,272 (84%) in the intervention group for the first, second, and third rounds of treatment, respectively. Use of LLINs was monitored in a subsample of children through weekly home visits. Similar proportions of children in the control and in the intervention group slept under an LLIN (92.7% versus 92.8%).

### Effect of IPTc on the Incidence of Clinical Malaria

169 (8.2%) children who had a positive RDT test were parasite negative by microscopy. Incidence of malaria defined as fever or history of fever with asexual parasitaemia ≥5,000/µl (primary endpoint) was estimated at 1.92 (95% CI 1.73–2.14) per child during the study period in Toeghin; 2.12 (95% CI 1.92–2.36) in Niou; 1.62 (95% CI 1.44–1.81) in Laye; and 1.65 (95% CI 1.47–1.84) in Sao. Overall the incidence of malaria was 1.3 (95% CI 1.11–1.53) in children aged 3–11 mo; 2.50 (95% CI 2.26–2.76) in children aged 12–23 mo; 2.56 (95% CI 2.31–2.83) in children aged 24–35 mo; 1.36 (95% CI 1.19–1.56) in children aged 36–47 mo; and 1.27 (95% CI 1.10–1.47) in children aged 48–59 mo. The incidence of clinical malaria was highest in children aged 12–35 mo (Table 2). 982 episodes of clinical malaria with asexual parasitaemia of 5,000/µl or more were recorded in children in the control group compared with 332 episodes in children in the intervention group. 523 children had one episode, 201 had two episodes, and 19 had three episodes in the control group. In the intervention group, 246 children had one episode, 43 had two episodes, and no child experienced three episodes. The unadjusted IRR was 0.30 (95% CI 0.26–0.34) (*p<*0.001), indicating a PE of 70%. Adjustment for age, sex, and village did not alter this estimate. There was strong evidence that the IRR varied with age (*p<*0.0001) with the effect of IPTc being strongest in the youngest children (3–23 mo). IPTc with SP + AQ was effective in reducing clinical malaria in the four study villages (Table 2). There was weak evidence to suggest that the IRR varied with village (*p = *0.06); the smallest protective effect was observed in the village of Sao and in the remaining villages, the protective effect was similar. Very few children were reported as not having used a net the previous night and so whether the effect of IPTc on clinical malaria varied with ITN use could not be examined.

**Table 2:** Effect of IPTc on the incidence of malaria, defined as fever or history of fever with 5,000 or more asexual forms of *P. falciparum* per µl, by age group and locality.

Age and Locality	Intervention (SP + AQ)		Control		Unadjusted IRR (95% CI)	*p*-Value	Adjusted IRR (95% CI)^a^	PE (1 − RR) (95%CI)	*p*-Value
	Episodes (Child Years)	Incidence Rate (95%CI)	Episodes (Child Years)	Incidence Rate (95% CI)					
***Age (mo)***									
3–23	107 (147.4)	0.73 (0.60–0.87)	439 (127.5)	3.44 (3.13–3.78)	0.21 (0.16–0.26)	<0.001	0.21 (0.16–0.26)	79 (74–84)	<0.001
24–59	225 (231.7)	0.97 (0.85–1.11)	543 (213.7)	2.54 (2.33–2.76)	0.38 (0.32–0.44)	<0.001	0.38 (0.32–0.44)	62 (56–68)	<0.001
Overall	332 (380.3)	0.87 (0.78–0.97)	982 (341.3)	2.88 (2.70–3.06)	0.30 (0.26–0.34)	<0.001	0.30 (0.26–0.34)	70 (66–74)	<0.001
***Locality***									
Toeghin	84 (96.4)	0.87 (0.70–1.08)	267 (85.6)	3.12 (2.77–3.52)	0.27 (0.21–0.36)	<0.001	0.27 (0.21–0.35)	73 (65–79)	<0.001
Niou	82 (87.0)	0.94 (0.76–1.17)	268 (77.4)	3.46 (3.07–3.90)	0.27 (0.21–0.35)	<0.001	0.26 (0.20–0.33)	74 (67 –80)	<0.001
Laye	73 (99.0)	0.74 (0.59–0.93)	231 (88.8)	2.60 (2.29–2.96)	0.28 (0.21–0.37)	<0.001	0.27 (0.21–0.36)	73(64–79)	<0.001
Sao	93 (97.8)	0.95 (0.78–1.16)	216 (89.5)	2.41 (2.11–2.75)	0.39 (0.30–0.50)	<0.001	0.39 (0.30–0.50)	61(50 –70)	<0.001

a IRRs were adjusted for age sex and village using random effect Cox Regression model.

PE was obtained using as follows. Note the incidence rates relate only to the three mo surveillance period are not an annual rate.

**Figure 2:**
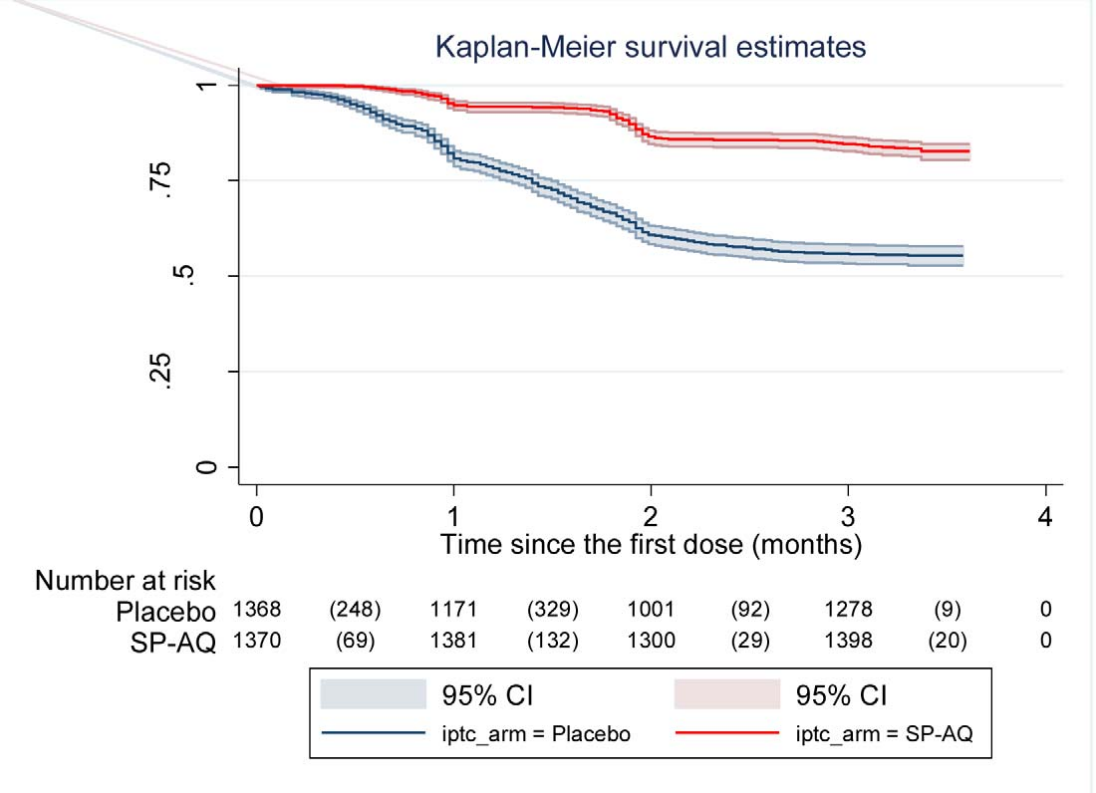
Time to first episode of clinical malaria defined as fever (≥37.5°C) or history of fever in the last 24 h and parasitaemia ≥5,000/µl in the intervention and control arms. Kaplan-Meier survival estimates with pointwise 95% confidence bands.

Administration of IPTc delayed the time until children experienced their first clinical attack of malaria defined as the presence of fever or a history of fever together with *P. falciparum* asexual parasitaemia at a density of 5,000 parasites/µl or more (*p<*0.0001) (Figure 2).

Analysis using the secondary endpoint definition of clinical malaria (fever or history of fever and the presence of asexual parasitaemia of any density) showed a similar reduction (IRR = 0.29; 95% CI 0.26–0.32) (*p<*0.001) to the reduction observed when clinical malaria was defined as fever or history of fever with the presence of at least 5,000 asexual parasites/µl. Severe malaria was observed in 13 children in the control group and in four children in the intervention group (PE = 69%; 95% CI 6%–90%) (*p = *0.04) (Table 3). The incidence of all-cause hospital admissions (20 cases in the intervention group compared with 37 in the control group) was 46% lower in the IPTc arm (95% CI 7%–69%) (*p = *0.03) (Table 3).

**Table 3:** Effects of IPTc on clinical malaria, defined as fever or history of fever with asexual *P. falciparum* parasitaemia of any density, severe malaria, and all-cause hospital admission.

Secondary Endpoint	Intervention (SP + AQ)			Control			Unadjusted IRR (95% CI)	*p*-Value	Adjusted IRR (95% CI)^a^	PE (95% CI)	*p*-Value
	*n* episodes	Days at Risk	Incidence Rate (95% CI)	*n* episodes	Days at Risk	Incidence Rate (95% CI)					
Clinical malaria	416	373.6	1.11 (1.00–1.22)	1,232	325.5	3.78 (3.58–4.00)	0.29 (0.26–0.32)	<0.001	0.29 (0.26–0.32)	71 (68–74)	<0.001
Severe malaria^b^	4	406.0	0.01 (0.004–0.026)	13	402.8	0.032 (0.019–0.056)	0.31 (0.10–0.94)	0.038	0.31 (0.10–0.94)	69 (6–90)	0.039
All-cause hospital admission	20	405.4	0.05 (0.03–0.08)	37	401.7	0.09 (0.07–0.13)	0.54 (0.31–0.92)	0.024	0.54 (0.31–0.93)	46 (7–69)	0.026
Deaths	3	406.5	7.4 † (2.4–22.9	7	403.4	17.4† (8.3–36.4)^c^	0.43 (0.11–1.64)	0.22	0.43 (0.11–1.64)	0.57 (−0.64 to 89)	0.22

a IRRs adjusted for age, sex, and village. Note the incidence rates relate only to the 3-mo surveillance period and are not an annual rate.

b Severe malaria was defined according to the WHO definition [17] “presence asexual forms of *P. falciparum* and any other of danger signs of severe malaria in the absence of any other cause of illness.”

c Death rates per 1,000 child year.

### Effect of IPTc on the Prevalence of Malaria Infection

The prevalence of malaria parasitaemia among children visited at home during the intervention period was lower in the intervention than in the control group (18.6% versus 45.8%) (PE = 59%, 95% CI 50%–67%) (*p<*0.001) (Table 4). A lower prevalence of gametocytes was observed in children who received the intervention than in children who did not (2.4% versus 10.3%; RR = 0.23; 95% CI 0.13–0.40) (*p<*0.001).

**Table 4:** Effect of IPTc on malaria infection, anaemia, and anthropometric indicators at the end of the malaria transmission season.

Secondary Endpoint	Intervention (SP+AQ)		Control		Unadjusted Analysis		Adjusted Analysis	
	Percent (*n*)	*n*	Percent (*n*)	*n*	RR (95% CI)	*p*-Value	RR (95% CI)	*p*-Value
***Weekly survey of malaria infection***								
Proportion with parasitaemia	18.6 (133)	715	45.8 (317)	692	0.40 (0.34–0.48)	<0.001	0.41 (0.33–0.50)	<0.001
***End of transmission survey***								
Proportion with parasitaemia	11.4 (164)	1,436	41.5 (594)	1,430	0.27 (0.23–0.32)	<0.001	0.27 (0.23–0.32)	<0.001
Proportion with anaemia (Hb<11 g/dl)	44.2 (638)	1,444	65.5 (944)	1,441	0.67 (0.63–0.72)	<0.001	0.67 (0.61–0.75)	<0.001
Proportion with moderate anaemia (Hb<8 g/dl)	2.7 (39)	1,444	6.2 (89)	1,441	0.44 (0.30–0.63)	<0.001	0.44 (0.30–0.64)	<0.001
Proportion with wasting	8.8 (122)	1,391	11.2 (156)	1,389	0.78 (0.62–0.98)	0.031	0.79 (0.65 –1.00)	0.049
Proportion with stunting	37.8 (526)	1,391	39.0 (542)	1,389	0.97 (0.88–1.06)	0.52	0.96 (0.85–1.08)	0.50
Proportion with underweight	20.8 (289)	1,391	24.7 (343)	1,389	0.84 (0.73–0.97)	0.015	0.84 (0.72–0.99)	0.034

RRs adjusted for age, sex and village using a Poisson regression generalized linear model (GLM). Wasting was defined as <−2 *z*-score weight for age. Stunting was define as <−2 *z*-score of height for age. Underweight was defined as <−2 *z*-score of weight for height.

The overall prevalence of malaria parasitaemia was 26.4% during the survey conducted at the end of the malaria transmission season (6 wk after the last course of IPTc treatment); 94.2% (714) and 1.2% (9) of these infections were single infections with *P. falciparum* and *P. malariae*, respectively, the remainder being mixed infections. The prevalence of malaria infection increased with age (*p<*0.001) and varied between villages (unpublished data). 6 wk after the last course of IPTc treatment, 11.4% (164) of children in the IPTc arm had malaria parasitaemia compared to 41.5% (594) in the control group (PE = 73%; 95% CI 68%–77%) (*p = *0.001). There was strong evidence that the effect of IPTc on malaria infection varied with age (*p = *0.003) with the effect being strongest in children aged 3–23 mo. The proportion of children who carried gametocytes postintervention as assessed by microscopy was also lower in children from the intervention arm than in the control arm (2.0% versus 9.3%) indicating a 79% (95% CI 68%–86%) reduction in the risk of gametocytes carriage in children who had received IPTc (*p<*0.001).

### Effect of IPTc on Anaemia

The prevalence of anaemia (Hb<11 g/dl) overall was high but decreased with age, from 70% (772) in children aged 3–23 mo to 45% (809) in children aged 24–59 mo (*p<*0.0001) but there was no evidence that it varied between village (*p = *0.42).

Hb concentration was higher in the intervention group than in the control group at the end of the malaria transmission season (mean Hb 11.01 g/dl [95% CI 10.94–11.08 g/dl] versus a mean of 10.35 g/dl [95% CI 10.27–0.42 g/dl]) (*p<*0.001). Anaemia (Hb<11 g/dl) was less common in children in the IPTc arm than in the control arm (RR = 0.67; PE = 33%, 95% CI 25–39) (*p<*0.001). 89 (6.2%) children in the control group had moderately severe anaemia (Hb<8 g/dl) versus 39 (2.7%) children in the intervention group (PE = 56%; 95% CI 36%–70%) (*p<*0.001). There were too few cases of severe anaemia (Hb<5 g/dl) to perform any meaningful comparative analysis between the intervention and control groups (zero and four cases, respectively).

### Effect of IPTc on Anthropometric Indicators

At the end of the malaria transmission season, IPTc was associated with a 21% reduction (95% CI 0%–35%) (*p = *0.05) in the risk of wasting (Table 4). Children in the intervention group were also less likely to be underweight (PE = 16%; 95% CI 1–28) (*p = *0.03). The prevalence of stunting was similar in the two groups (*p = *0.5). Comparison of the mean weight gain in the two treatment arms using data from children who were present both at the baseline and postintervention survey indicated greater weight gain in the intervention than in the control group (0.72 kg versus 0.57 kg) (*p<*0.0001). The effect of IPTc on clinical malaria varied in stunted and nonstunted children (*p = *0.0001) with PEs of 62% (95% CI 54%–69%) and 78% (95% CI 73%–81%), respectively. No evidence of an effect modification was observed for underweight (*p = *0.18) or wasting (*p = *0.07).

### Effect of IPTc on Antimalarial Drug Resistance

The prevalence of genetic markers of resistance to SP and AQ was assessed before the intervention in children 3–59 mo old who were not enrolled in the trial but who lived in the same communities. The proportions of children carrying parasites with triple *dhfr* mutations at codons 51, 59, and 108 associated with resistance to pyrimethamine or the triple *dhfr* plus single *dhps* mutation (mutation at codon 437), associated with resistance to sulphadoxine, were estimated at 32.6% and 25%, respectively (Table 5). No child carried an infection with triple *dhfr* and *dhps* mutations at codons 540 and 437. There was no evidence of a difference in the proportions of children who carried mutant parasites between the intervention and control groups during the postintervention survey. However, there was an overall increase between baseline and postintervention surveys in the proportions of children with triple *dhfr* mutations only (*p<*0.001) and triple *dhfr* plus a single *dhps* (codon 437) mutation (*p = *0.001). The proportions of children harbouring parasites with the *pfcrt*-76 or *pfmdr1*-86 mutations were similar in the control and intervention arms and did not increase postintervention as compared to baseline.

**Table 5:** Percentage of children carrying parasites harbouring genetic mutation associated with resistance to SP and AQ at baseline and 6 wk postintervention in intervention and control arms.

Genetic Mutation	Baseline, *n = *132, Percent (*n*)	Postintervention			
		IPTc, *n = *114, Percent (*n*)	Placebo, *n = *122, Percent (*n*)	Overall postintervention, *n = *236, Percent (*n*)	Baseline versus postintervention, *p*-Value
Triple *dhfr* mutation (51-59-108)	32.6 (43)	50 (57)	53.3 (65)	51.7 (122)	<0.001
Triple *dhfr* single *dhps* (437)	25.0 (33)	40.4 (46)	44.3 (54)	42.4 (100)	0.001
Triple *dhfr*/double *dhps* (437–540)	0 (0)	0.9 (1)	0.0 (0)	0.4 (1)	0.45
*Pfcrt*-76	63.9 (83)	60.5 (69)	61.5 (75)	61.0 (144)	0.72
*Pfcrt*-76/*pfmdr1*-86	20.5 (27)	19.3 (22)	19.7 (24)	19.5 (46)	0.82

A small *in vivo* study conducted in asymptomatic children resident in the study area in the year after the intervention study confirmed the efficacy of the SP + AQ combination in clearing asymptomatic *P. falciparum* infections (Text S4).

### Adverse Events

Ten deaths were observed during the intervention period, seven in children in the control group and three in children in the intervention group. Verbal autopsies suggested that four deaths (three deaths in the control group) were associated with malaria, three with acute diarrhoea and malnutrition, two with diarrhoea only, and that one death was due to pneumonia.

No drug-related serious adverse events were observed during the follow-up period. The risk of itching, skin rash, diarrhoea, drowsiness, or loss of appetite did not differ between children who received SP plus AQ and those who received placebo (Table 6). The proportion of children who vomited at least once during the three courses of treatment was higher in children who received SP + AQ, 368/1,339 (27.6%), than in children who received placebos of SP plus AQ, 122/1,257 (9.7%). Amongst children who received SP plus AQ, those aged 3–11 mo were at highest risk of vomiting and the risk of vomiting decreased with age (Table 7). The proportions of children who vomited during the first, second, and third round of treatment were 17% (197), 15% (184), and 13% (160), respectively, in children who received SP + AQ. The RR for vomiting remained similar between the first (RR = 2.9), the second (RR = 3.0), and the third (RR = 3.3) course of treatment in the intervention group when compared to the control group.

**Table 6:** Percentage of children with adverse events on at least one occasion during the three rounds of treatment in intervention and control arms.

Adverse Event	IPTc	Placebo	RRs (95% CI)	*p*-Value
	Percent (*n/n*)	Percent (*n/n*)		
Fever	13.4 (179/1,339)	14.9 (187/1,257)	0.90 (0.74–1.11)	0.33
Vomiting	27.6 (368/1,339)	9.7 (122/1,257)	2.83 (2.31–3.47)	<0.001
Drowsiness	0.1 (1/1,339)	0.1 (1/1,257)	0.94 (0.06–15.0)	0.96
Itching	2.2 (30/1,339)	2.4 (30/1,257)	0.94 0.56–1.56)	0.80
Diarrhoea	7.2 (96/1,339)	7.0 (88/1,257)	1.02 (0.77–1.37)	0.86
Skin rash	1.5 (20/1,339)	1.6 (20/1,257)	0.94 (0.50–1.74)	0.84
Coughing	5.2 (70/1,339)	5.9 (74/1,257)	0.92 (0.66–1.27)	0.60
Loss of appetite	1.4 (5/1,339)	1.3 (3/1,257)	1.51 (0.36–6.34)	0.57

**Table 7:** Risk of vomiting by age in children who received SP + AQ.

Age group (mo)	Percent (*n/m*)	RR (95% CI)	*p*-Value
3–11	54.5 (114/209)	9.9 (5.6–17.2)	<0.001
12–24	38.7 (117/302)	7.0 (4.0–12.2)	<0.001
24–35	28.6 (75/262)	5.2 (2.92–9.2)	<0.001
36–47	14.5 (48/309)	2.8 (1.5–5.1)	0.001
48–59	5.5 (14/253)	1	—

## Discussion

To the best of our knowledge, this is the first study to test if IPTc provides protection against malaria in children who are already protected by an ITN. Our results show conclusively that IPTc does provide substantial protection against clinical malaria episodes, severe malaria, and all-cause hospital admissions in children using an ITN. The primary role of ITNs is to prevent mosquito bites, thus reducing the risk of malaria infection, whereas IPTc clears existing infections and has a prophylactic effect preventing new blood stage infections. The high PE of IPTc in children using ITNs may partly reflect protection from infections acquired because of exposure to mosquitoes at night outside sleeping hours. Coverage of ITNs was low among older children and adults in the study area; the absolute reduction in malaria incidence due to IPTc may have been less if coverage of ITNs in the population as a whole had been higher. A similar additional effect of combining chemoprevention with ITNs was observed in a community randomised study of ITNs and chemoprophylaxis with Maloprim (dapsone-pyrimethamine) given every 2 wk in Sierra Leone [23]. In this study, ITNs and chemoprophylaxis alone were associated with 49% and 42% protection against malaria, respectively, whereas the combined effect of the two interventions was 72% (95% CI 67%–76%). Consistent findings have also been reported from The Gambia, where seasonal chemoprophylaxis with Maloprim protected children from malaria attacks in villages where ITNs were being used [24].

The efficacy of IPTc against clinical attacks of malaria was a little less in our study than that seen in Senegal [6] but very similar to that obtained in Mali [7] and Ghana [8] in populations with a low use of ITNs. Varying efficacy of IPTc between trials may be explained by a number of factors including transmission intensity and nutritional status. Our results showed that IPTc was more beneficial to children who were not stunted. A previous study by Danquah and collaborators [25] had reported that IPT of malaria in infants was less effective in malnourished than in non-malnourished infants. In our study, the impact of IPTc was more marked in children less than 24 mo, who have yet to achieve significant acquired immunity to malaria, than in older children, a similar pattern to that observed in Senegal [6]. A total of 3,756 courses of IPT treatment were administered in the SP + AQ arm, and 650 cases of malaria were averted, thus 5.8 IPT administrations were needed to prevent one episode of malaria.

The reduction in the prevalence of anaemia seen in our study is consistent with the results of the study conducted in Hohoe, Ghana [8] where a 45% reduction in anaemia was observed in children who received IPTc with AS plus AQ monthly, and a 30% reduction was seen in children who received bimonthly IPTc with SP or AS plus AQ. However, IPTc with AS plus SP did not result in a detectable reduction in anaemia in Senegal [6]. It is likely that the fraction of anaemia attributable to malaria differs between settings and this fraction is likely to be lower in areas with lower transmission intensity such as Senegal. Modest reductions were observed in the proportion of wasted and underweight children after IPTc administration, but there was no effect on stunting. The lack of an effect on stunting is perhaps not surprising as this is generally held to be a measure of chronic undernutrition, which is less likely to change substantially over the period of follow-up reported in this paper than indicators of acute undernutrition. IPTc increased the mean weight gain during the rainy season as previously reported in a study that also demonstrated an increase in subcutaneous fat reserves in Senegalese children who received IPTc with SP plus AS [26].

The study was not powered to detect an impact on cases of severe malaria, hospital admissions, or deaths. However, encouraging results were found with a reduction in deaths, overall hospital admissions, and severe malaria in children who received IPTc with differences in cases of severe malaria and hospital admissions being statistically significant. Thus, it seems likely that if IPTc was widely deployed in areas with a similar pattern of malaria transmission to that of the study area it would have a significant impact on severe morbidity and mortality from malaria. This supposition is supported by the results of an earlier study conducted in The Gambia [27], which showed that chemoprophylaxis with Maloprim given fortnightly during the rainy season reduced overall mortality in children aged less than 5 y by about 40%.

We monitored adverse events over 4 d during each course of treatment and detected no serious adverse events related to the study drugs, which were reasonably well tolerated and safe. However, we observed a higher risk of vomiting in the intervention group, as has been reported in previous IPTc studies that used drug combinations containing AQ [Bibr pmed.1000408-Kweku1] [Bibr pmed.1000408-Sokhna1] [8,13,28]. Unlike the Hohoe study, the frequency of vomiting did not decrease with subsequent courses of IPTc. Our data showed a considerably higher risk of vomiting in younger children. Vomiting induced by AQ was not a significant deterrent to the use of SP plus AQ because compliance with the full course of treatment was higher in children who received IPTc than in those who received placebo. Nevertheless, the acceptability of SP plus AQ for IPTc would be improved if this problem could be overcome. There are at least two possible ways that this might be accomplished. A recent study undertaken in Senegal showed that the frequency of vomiting following the use of AQ containing combinations for IPTc is dose dependent [29]. Thus, one possibility would be to adjust the AQ content of tablets used for IPTc, allowing the optimum spread of dose per kg body weight for each weight group and ensuring that as few as possible children are overdosed. A second approach would be to produce a formulation of AQ that was more palatable than standard tablets.

A number of concerns have been raised about the adoption of IPTc as a malaria control strategy. These include the possibility that IPTc will encourage the spread of resistance to the drugs used for IPTc. We observed an increase in the proportion of children harbouring genetic mutations associated with resistance to SP at the end of the malaria transmission season compared with the preintervention period but not in the prevalence of genetic mutations associated with resistance to chloroquine. However, in contrast to the findings of a study conducted in Senegal [6], we found no difference in the prevalence of resistance markers between intervention and control groups. This difference between studies might be due to the higher level of malaria transmission in the study area compared with Senegal, resulting in a higher rate of exchange of parasite strains between the two treatment groups and the rest of the population. An overall increase in the prevalence of drug resistance markers over the course of the malaria transmission season has been seen previously in areas of seasonal malaria transmission in the absence of any chemopreventive strategy [30], probably reflecting the selection pressure of an overall increase in the use of antimalarials for the treatment of febrile illnesses at this time of the year. However, we cannot exclude the possibility that the use of IPTc made some contribution to the overall increase in the prevalence of *dhfr* resistance markers seen at the end of the transmission season and sustained implementation of IPTc would inevitably increase drug pressure, a risk that would need to be balanced against the marked benefit that can result from this intervention. This risk can be reduced by using a drug combination rather than a single drug for IPTc and by using a different drug combination for IPTc than the one used for first-line treatment of symptomatic malaria.

Another concern is that IPTc might interfere with the development of naturally acquired immunity to malaria, leading to an increase in the incidence of malaria in children after they move out of the age range in which IPTc is given. Administration of IPTc for 1 y did not lead to an increase in the incidence of clinical attacks of malaria in the following year in children in Senegal [6] or in older children in Ghana [8]. Nevertheless, we are investigating this possibility in the current study and the surveillance procedure described in this paper was reestablished during the 2009 malaria transmission season to look for any evidence of “rebound” malaria. Even if no increase in risk is found, this does not exclude the possibility that IPTc could significantly impair the development of natural immunity if administered to children for several consecutive years and this would have to be monitored carefully if IPTc is implemented as a malaria control strategy.

Another major concern is whether IPTc could be delivered on a large scale. As this was an efficacy trial, medications were given under the control of project staff and this study did not address the issue of the implementability of IPTc. This issue has been addressed in a previous study undertaken in Ghana [31] and a study that has investigated two possible modes of delivery in The Gambia, use of immunisation trekking teams or community volunteers is described in an accompanying paper [32]. A study of the feasibility and safety of large scale implementation of IPTc with SP + AQ is currently underway in Senegal.

A notable feature of this study was the high incidence of malaria in children in the control group who slept under an LLIN: 982 of 1,505 children experienced a clinical attack during the 3-mo observation period that corresponded to the peak malaria transmission season. A number of possible reasons for this result have been considered. Home visits indicated that more than 90% of the children had used their LLIN the previous night so that failure to use a bednet is unlikely to have been a major factor. It is possible that some children experienced mosquito bites before they retired to bed but this is less likely to be a problem than in older children or adults. LLINs (PermaNet) were obtained from a well-established manufacturer (Vestergaard). Checks on the deltamethrin content of ITNs obtained at the end of the malaria transmission season showed protective levels of deltamethrin in most samples and this was confirmed by bioassays (Text S7). However, some *kdr-*mediated resistance to pyrethroids was found (Text S7). Although the entomological inoculation rate (EIR) (34 infective bites per person per year) was significantly less than had been recorded in the same area 6 y previously, perhaps in part due to the use of LLINs, this was sufficient to sustain a high clinical attack rate in young children. LLINs were provided only to children in the study so it is likely that, counting these children, their mothers, and their older siblings, only about a third of the population were protected by an LLIN. To obtain maximum benefit from ITNs in high transmission settings such as this, universal coverage with ITNs is probably required.

### Conclusion

IPT of malaria with SP plus AQ was safe and provided substantial additional protection against severe and uncomplicated malaria to children who slept under an LLIN. There is now strong evidence to support the integration of IPTc into malaria control strategies in areas of seasonal malaria transmission. Further research is needed to identify alternative drugs as the long-term use of SP for IPTc is uncertain.

## Supporting Information

Text S1 Study protocol.
(0.19 MB PDF)

Text S2 Protocol amendment.
(0.11 MB PDF)

Text S3 CONSORT checklist.
(0.09 MB PDF)

Text S4 In vivo efficacy of the SP + AQ combination. Results of a pilot study of in vivo efficacy of SP plus AQ in children with uncomplicated malaria in the study area.
(0.51 MB PDF)

Text S5 In vivo efficacy of the SP + AQ combination. Results of an in vivo study of the efficacy of SP plus AQ in clearing malaria infection in children with asymptomatic malaria. The study was conducted 1 y after the intervention.
(0.44 MB PDF)

Text S6 External quality assurance of malaria microscopic diagnosis. Report of external quality control of malaria microscopic diagnosis.
(0.10 MB PDF)

Text S7 Entomological investigations. Reports on malaria transmission intensity estimation, bioassays, vector resistance to insecticide, and concentration of insecticide on LLINs.
(0.46 MB PDF)

## Abbreviations

AQ: amodiaquine
AS: artesunate
CI: confidence interval
Hb: haemoglobin
IPT: intermittent preventive treatment of malaria
IPTc: intermittent preventive treatment of malaria in children
IPTi: intermittent preventive treatment in infants
IRR: incidence rate ratio: ITN, insecticide-treated net
LLIN: long-lasting insecticide-treated bednet
PE: protective efficacy
RDT: rapid diagnostic test
RR: risk ratio
SP: sulphadoxine pyrimethamine
