# Supplementary material for: Intermittent Preventive Treatment of Malaria Provides Substantial Protection against Malaria in Children Already Protected by an Insecticide-Treated Bednet in Burkina Faso: A Randomised, Double-Blind, Placebo-Controlled Trial
Source: PLoS Med. 2011 Feb 1;8(2):e1000408. doi: 10.1371/journal.pmed.1000408 (PMC3032552; doi:10.1371/journal.pmed.1000408)
Supplement: Text S4 — In vivo efficacy of the SP + AQ combination. Results of a pilot study of in vivo efficacy of SP plus AQ in children with uncomplicated malaria in the study area. (0.51 MB PDF) [file pmed.1000408.s004.pdf]

SUPPORTING INFORMATION – TEXT S4

In vivo efficacy of the sulphadoxine/pyrimethamine (SP) + amodoaquine (AQ) combination against symptomatic *Plasmodium falciparum* malaria in the study area

Introduction

A pilot study was conducted prior to the IPTc trial to assess the *in vivo* efficacy of the sulphadoxine pyrimethamine (SP) plus amodiaquine (AQ) combination over 28 days for the treatment of uncomplicated malaria and to evaluate its potential for intermittent preventive treatment (IPT) of malaria in children in Burkina Faso.

Methods

The study was conducted in 6 health centres of the Boussé health district (Laye, Niou, Sao, Toeghin, Goldmidou and Likenkelsé) in the malaria transmission season of 2006. Children aged 6-59 months with fever were screened for enrolment. Inclusion and exclusion criteria were based on the WHO standard protocol for the monitoring of antimalarial drug resistance [1]. Eligible children with uncomplicated malaria were treated with standard doses (25 mg/kg) of SP (single dose) plus AQ (10mg/kg daily for 3 days) and invited to return to the health centre to complete treatment on the following 2 days, on days 7, 14, 21 and 28 for the monitoring of treatment outcome and on any other time in between if they felt sick. Blood smears and filter paper blood spots were collected at enrolment and on days 7, 14, 21 and 28. Blood smears were stained with Giemsa and examined by two laboratory technicians. In case of a discrepancy the slides were examined by a third laboratory technician.

Results

Between October and November 2006, 214 children were screened and 137 were enrolled. Two of the enrolled children withdrew consent on day 1, one child received concomitant treatment with quinine and another child died, leaving 133 children for inclusion in the per protocol analysis. The numbers of children with treatment failure (ETF+LTF+LPF) were 5 and 11 on days 14 and 28 respectively. The efficacy (PCR-uncorrected) of SP+AQ for the treatment of uncomplicated malaria in the study area was therefore 96.2% (95%CI: 91.4% – 98.8%) on day 14 and 91.7% (95%CI: 85.6% - 95.8%) on day 28.

Conclusion

The study showed that SP+AQ combination was effective for the treatment of uncomplicated malaria, confirming findings from a previous study conducted in southern Burkina Faso which reported a PCR-corrected adequate clinical and parasitological response of 95% [2] and that the SP + AQ combination was, therefore a suitable combination for us ein the IPTc trial.

References

1. World Health Organisation. Monitoring antimalarial drug resistance (2002). WHO/CDS/RBM/2002.39.
2. Zongo I, Dorsey G, Rouamba N, Dokomajilar C et al. (2005). Amodiaquine, sulfadoxine-pyrimethamine, combination therapy for the uncomplicated falciparum malaria: randomised controlled trial from Burkina Faso. Am. J Trop. Med Hyg. **75**: 826-832
