# Supplementary material for: Intermittent Preventive Treatment of Malaria Provides Substantial Protection against Malaria in Children Already Protected by an Insecticide-Treated Bednet in Burkina Faso: A Randomised, Double-Blind, Placebo-Controlled Trial
Source: PLoS Med. 2011 Feb 1;8(2):e1000408. doi: 10.1371/journal.pmed.1000408 (PMC3032552; doi:10.1371/journal.pmed.1000408)
Supplement: Text S5 — In vivo efficacy of the SP + AQ combination. Results of an in vivo study of the efficacy of SP plus AQ in clearing malaria infection in children with asymptomatic malaria. The study was conducted 1 y after the intervention. (0.44 MB PDF) [file pmed.1000408.s005.pdf]

SUPPORTING INFORMATION – TEXT S5

In vivo efficacy of the sulphadoxine/pyrimethamine (SP) + amodoaquine (AQ) combination against asymptomatic *Plasmodium falciparum* malaria in the study area.

Introduction

In order to confirm the *in vivo* efficacy of the SP + AQ combination used in the IPTc study in eliminating malaria parasitaemia in asymptomatic children, a small observational study was undertaken in asymptomatic children aged 15-71 months with *Plasmodium falciparum* parasitaemia resident in the study area in 2009, the year after the IPTc intervention had been conducted. These children were aged 3-59 months at the beginning of the intervention in 2008.

Methods

Children were screened for inclusion in the study during a cross-sectional survey in November 2009. Blood films were obtained from children with a positive rapid Rapid Diagnostic test (RDT , OPTIMAL IT®; Diamed AG, Cressier FR, Switzerland) and screened for malaria parasitaemia. Asymptomatic children with *P. falciparum* parasitaemia were eligible for inclusion in the trial. After informed consent had been obtained, eligible children were given supervised treatment with SP (25mg/kg as a single dose) and AQ (10 mg/kg daily for 3 days) and appointments were given on days 1 and 2 for completion of treatment and on days 7, 14, 21 and 28 for the monitoring of parasite clearance. Thick and thin blood smears were prepared at enrollment, on days 7, 14, 21, 28 and on any unscheduled visit. Blood films were examined by two independent laboratory technicians and malaria parasites were counted against 200 white blood cells. Slides with discrepant positive and negative results or with parasite counts which differed by more than 30% were re-examined. Failure to clear parasite was defined as the presence of asexual parasite at any density from day 4 to day 28, regardless of body temperature.

Results

Two-hundred and fifty-two children with asymptomatic malaria were selected for inclusion in the study based on RDT results. Fourteen children were excluded because of a broken or lost slide (6) or a negative blood film (8). Two hundred and seventeen children received SP+AQ and provided finger prick blood samples for the assessment of parasitaemia on days 7, 14, 21 and 28. Parasitaemia could not be assessed for 21 children on at least one of the specified assessment days because they did not attend or the slide could not be found. The efficacy of SP+AQ in clearing *P falciparum* in asymptomatic children was 99.5% (216/217) and 99% (215/217) on days 14 and 28 respectively.

Conclusion

The results of this small observational study confirm the *in vivo* efficacy of the SP + AQ combination in clearing malaria parasitaemia from asymptomatic children in the study area. As noted in previous studies of intermittent preventive treatment in pregnant women and in infants, drugs or drug combination may retain high efficacy in clearing low density parasitaemia from asymptomatic subjects after they have lost some of their efficacy in treating clinical infections in young children.
