# Supplementary material for: Intermittent Preventive Treatment of Malaria Provides Substantial Protection against Malaria in Children Already Protected by an Insecticide-Treated Bednet in Burkina Faso: A Randomised, Double-Blind, Placebo-Controlled Trial
Source: PLoS Med. 2011 Feb 1;8(2):e1000408. doi: 10.1371/journal.pmed.1000408 (PMC3032552; doi:10.1371/journal.pmed.1000408)
Supplement: Text S7 — Entomological investigations. Reports on malaria transmission intensity estimation, bioassays, vector resistance to insecticide, and concentration of insecticide on LLINs. (0.46 MB PDF) [file pmed.1000408.s007.pdf]

SUPPORTING INFORMATION –TEXT S7

Entomological investigations

A surprising feature of the study described in this paper is the high malaria attack rate in children who slept under an ITN and who did not receive IPTc. This may have been due in part to the fact ITNs were given only to children in the trial and not to the whole community. However, a number of other studies were undertaken to explore this finding further.

1. Entomological Inoculation Rate in the study area

Introduction

A small study was undertaken in villages involved in the IPTc study from August to December 2009 to estimate the Entomological Inoculation Rate (EIR), perform bioassays, estimate the prevalence of genetic mutations associated with vector resistance to insecticide and determine the concentration of deltamethrin on long-lasting insecticide treated nets (LLINs) given to children.

Methods

The list of children from the census database was used to sample compounds for mosquito catches. Selected compounds were visited by trained fieldworkers who picked one house at random for mosquito catches. Mosquito catches were performed indoors using CDC light traps (J. W. Hock Co, Gainesville, Florida, USA) which were positioned from 6 pm to 6 am near a human volunteer sleeping under an untreated bed net. Catches were performed from August to December 2009 on two consecutives nights in 10 houses per week per village (340 catches were performed per village during the study period).

Heads and thoraces of mosquitoes caught in the light traps were tested for *Plasmodium falciparum* circumsporozoite protein (CSP) by performing duplicate ELISA essays on each mosquito specimen. Each mosquito specimen was tested separately. Only tests which gave an optical density of more than 2 standard deviations of the optical density of negative samples were considered CSP positive. EIR was calculated by multiplying the sporozoite rate by the geometric mean of vector density per person.

Results

From mid August to mid December 2009, 340 light trap catches were set in each of the 4 villages involved in the IPTc trial (1360 in total). In total, 1470 mosquitoes were caught during the survey; the largest number of mosquitoes was caught in Sao (767) followed by Laye (392). The mean vector

agressivity per person per night was 1.10. 1193 (81%) mosquitoes were tested by ELISA for the presence of CSP and 102 mosquitoes carried *P falciparum* sporozoites indicating a mean sporozoite rate of 9% in the study area. Based on the findings, the EIR for the study area as a whole was estimated to be 34 infective bites per person per year with the highest EIR being observed in Sao (EIR=74) and lowest EIR in Niou (EIR=11).

Conclusions

The results obtained from this small study suggested that the EIR in the study area was 34 infective bites per person per year, a substantially lower figure than the figure of 173 recorded in the area in 2002 when no major intervention with ITNs was in place. Why this fall has occurred is not apparent, but it is possible that partial coverage with LLINs during the study may have played a role. Nevertheless, it indicates that there is still substantial malaria transmission occurring in the study villages, concentrated in a few months of the year when the inoculation rate is high. These findings are compatible with an incidence rate of 3-4 clinical attacks of malaria per year in young children and thus with an attack rate of 1-2 clinical attacks per year in children sleeping under an ITN, as observed in the study.

2. Deltamethrin content of treated nets

Introduction

At the beginning of the study, the family of each study child was provided with an LLIN (PermaNet® Vestergaard) to be used by that child during the course of the study and with instructions on how to use it. To confirm that the ITNs used in the IPTc study had retained chemical activity and were thus likely to be effective, an analysis was made of the deltamethrin content of a random sample of nets used by study children and on unused nets kept in the store.

Methods

During the entomological survey in 2009, in each of the 4 study villages, 10 LLINs which were being used by children enrolled in the IPTc trial were randomly selected and 2 pieces (25x25 cm) were cuts from 2 sides of each net. Pieces of nets of the same size were also obtained from 40 unused LLINs which were kept in the store for the replacement of damaged nets. Deltamethrin was extracted from the netting samples with solvents and the deltametrhin content of the extract determined by high performance liquid chromatography as previously described [1]. Assays were undertaken in duplicate on two samples from each net.

Results

Deltamethrin was detected in all the netting samples tested, confirming that study children had been using an ITN. However, a wide range of values of estimated deltamethrin content on the netted materials was obtained which varied from less than 1mg/m^2 to 45 mg/m^2 between nets. The mean concentration for the 40 tested LLINs which were being used was 11.2 mg/m^2. Mean concentrations varied between villages with means of 9.8 mg/m^2 in Toeghin, 10.5 mg/m^2 in Niou, 11.8 mg/m^2 in Laye and 12.5 mg/m^2 in SAO.. A much higher concentration of insecticide was found (35.5 mg/m^2) in unused LLINs, although below the 55 mg/m^2 mentioned on the packaging of the LLINs, indicating that they an appropriate concentration of insecticide was present on the nets at the time when they were given to the children. The lower concentration of insecticide on used nets as compared to unused ones suggests that used nets had been washed several times since installation.

Conclusions

The results confirmed that the nets used in the study had been treated with an appropriate concentration of deltamethrin. Lower concentrations than expected were found in some samples which may have been due to the fact that the nets had been washed several times, as is common practice in the study area mainly because of dust and cooking smoke.

3. Pyrethroid resistance

Introduction

A possible explanation for the apparent lack of efficacy of the LLINs is that anopheline mosquitoes in the study area have become resistant to pyrethroids and studies were carried out to explore this possibility.

Methods

Mosquito larvae were collected from mosquito breeding sites identified in the study area and kept at the CNRFP insectary to grow. Unfed female mosquitoes obtained from the larvae were then used for bioassay tests following the WHO standard protocol [2]. At least 100 females were exposed to deltamethrin (0.05%) or DDT (4%) per village. In addition, female mosquitoes from larvae collected in the study area were also exposed to pieces of netting cut from used and unused LLINs, as described in the previous section, for 3 minutes using the WHO protocol [2]. Mortality was assessed after 24 hours of exposure. An equal number of surviving and dead mosquitoes exposed to the

pieces of LLINs were tested for the presence of the kdr gene using the method described by Ranson et al [3].

Results

411 mosquitoes caught in the study area were tested (at least 100 mosquitoes per village) against deltamethrin 0.05% and the same number of mosquitoes was tested against DDT (4%) impregnated papers. Susceptibility of mosquitoes assessed using WHO impregnated papers of deltamethrin 0.05% varied between 75% (Laye) to 95% (Niou) after 24 hours of exposure. Susceptibility to DDT (4%) varied between 70% and 82%.

2450 female mosquitoes obtained from mosquito larvae collected in the four study villages were tested against pieces of netting obtained from used and unused LLINs. The efficacy of used LLINs in killing mosquitoes was 47% (95%CI: 32%-61%) in Toeghin, 57% (95%CI: 42%-73%) in Sao, 58% (95%CI: 48%-66%) in Laye and 59% (95%CI: 48% - 70%) in Niou and no significant differences were observed between villages. A similar result was observed with unused LLINs (56%; 95% CI: 39%-73%).

1640 mosquitoes were analysed for the presence of the kdr gene. 731 of these mosquitoes were fully resistant (48.5%) and 17 (1.1%) were mixed. The frequency of the *kdr* gene in the study area was 50% (95%C.I: 47.1% – 52.2%) ranging from 0.38 (0.32 – 0.44) in Niou to 0.59 (95%: 0.48-0.69) in Laye. No study of the frequency of the kdr gene had previously been conducted in the study area. However, studies in others areas of Burkina Faso with similar ecological conditions had reported similar frequencies of the kdr gene which varied from 0.047 to 0.54. Much higher frequencies of kdr have been found in areas with rice irrigation [4].

Conclusion

The results of this small study show that some resistance to deltametrhin is present in the study area and this may have contributed to the high attack rate in children who slept under a new LLIN.

References

1. Yates A, N'Guessan R, Kaur H, Akogbeto M, Rowland M. Evaluation of KO-Tab 1-2-3 [R] : a wash resistant 'dip-it-yourself' insecticide formulation for long-lasting treatment of mosquito nets (2005). *Malar J* : 4:52.
2. WHO: Test procedures for insecticide resistance monitoring in malaria vectors, bio-efficacy and persistence of insecticides on treated surfaces. *WHO/CDS/CPC/MAL/9812* 1998

3. Ranson H, Jensen B, Vulule JM, Wang X, Hemingway J, Collins FH. Identification of a point mutation in the voltage-gated sodium channel gene of Kenyan *Anopheles gambiae* associated with resistance to DDT and pyrethroids (2000). *Insect Mol Biol*. 9 (5):491-7

4. Dabiré KR, Diabaté A, Namountougou M, Toé KH, Ouari A, Kengne P, Bass C, Baldet T. (2009). Distribution of pyrethroid and DDT resistance and the L1014F kdr mutation in *Anopheles gambiae s.l*. from Burkina Faso (West Africa). *Trans R Soc Trop Med Hyg*. 103(11):1113-20.
